# Danube as a symbol of Europe. Perception of the river from varied geographical perspectives

**DOI:** 10.1371/journal.pone.0260848

**Published:** 2021-12-02

**Authors:** Tomasz Padło, Paweł Struś, Agnieszka Gil

**Affiliations:** Institute of Geography, Pedagogical University of Krakow, Krakow, Poland; Szechenyi Istvan University: Szechenyi Istvan Egyetem, HUNGARY

## Abstract

The Danube is promoted as a pan-European river, what can be justified for instance by the vast range of its drainage basin, covering 19 countries on both sides of the historical border diving Eastern and Western Europe. Differentiation of imaginations of Danube course from the perspective of 7 European cities, based on research covering 1577 respondents, conducted between 2005–2007 and 2016–2018 has been presented in the paper. Maps presenting the generalized imagination of river course have been generated for each city. It has been proved that in spite of substantial political, economical and symbolical importance of this river for big part of Europe, the course of Danube remains unknown for inhabitants of its Western part, in parallel to more correct recognition of the river by students from Eastern Europe. It has been shown that the perception does not change despite the progressing integration processes.

## Introduction

The Danube occupies an important place in the European space not only due to its length or the surface of its drainage basin but mostly thanks to its pan-European character in economical and cultural terms. 10 countries it flows through and the further 9 ones lying in its drainage basin make Danube a kind of symbol of international cooperation and reconciliation between the East and the West of Europe. The geopolitical, economical and cultural importance of the Danube for Europe has been recognized for a long time [1–3]; nowadays it is more and more often promoted as a symbol of Europe [4], what can be exemplified i.a. by cooperation of Danube countries within EUSDR (EU Strategy for the Danube Region) program which, besides of economical and cultural integration, aims at promoting EU values beyond its borders [5,6]. The researchers from the East of the continent perceive the Danube as the economical and cultural axis of Europe and the symbolical bridge linking the European family and the symbol of European integration [7]. Nevertheless, the importance of the Danube significantly exceeds the limits of Eastern Europe; among its biggest admirers one may find i.a. Magris and Canetti [8,9].

The Danube has also become a symbol of significant Central European or Balkan cities, like Vienna, Budapest or Belgrade. The name of Vienna in Slovenian is Danube, which shows the connection of Vienna with the Danube in the minds of the nations. Its importance for the region is also demonstrated by the concept of Donauraum, defining the community identity of the Danube regions [10]. The Danube is included in the anthems of 5 countries, which proves its importance [11], while at the same time occupying a special place in the space of Europe, as it could never become a national river, like the Vistula or the Vltava. Its essence is the transnationality [11].

This symbolical importance of the Danube induced the authors to question about the universality of perception of the river from various European perspectives and about the change of the perception of its course during the unification of the Danube countries, incl. within the European Union. Is the knowledge of its course so much common in the context of importance attributed to it, and is the Danube so well recognized that a European brand can be generated basing on the river, as proposed by Koev [12]? Or, in contrary, is the perception of the Danube course restricted to respondents from Danube countries, as it may be suggested by ethnocentrism visible in our imagination of the space [13]? And finally, do the European integration and the expansion of the European Union to the East result in a better perception of the river course? The answer to the question about the perception of the Danube by the inhabitants of Europe was to be given by the analysis of handwritten drawings of the Danube course, made by respondents from various European cities during the years of the European Union expansion to the East, and a decade later, when the new member states already settled within the originally Western-only structures.

## Literature review

The defining basis of the research is the concept of The image of the perceived space stored in the human mind creates the so-called cognitive map [14], referred to as an individual’s knowledge of spatial and environmental relations, as well as the cognitive processes related to coding and reconstructing in-formation which makes up this knowledge [15,16].

The research was based on the methodology taken from studies dedicated to the perception of space, using the methods of sketch map analysis. Proposals for researching the perception of the space and the space experienced by humans itself appeared in geography in the 1940s, when Tolman for the first time defined "cognitive mapping" as the way people think about space [17], and Wright [18] pointed out at the possibility of developing the interests and the research of the geographers onto the image of the world contained in human imaginations, considering them to be the most fascinating *terrae incognitae* of our times. The breakthrough in the research was brought by the 1960s and the works of the urban planner Kevin Lynch [19] on the perception of urban space, which initiated the research on imaginary maps resulting from perception of the space and developed methods which are in use in the study of the perception of space [20] up till today In the following decades, this trend of research was continued in various parts of the world and at various spatial scales. The preferences of the place of residence of British school graduates were studied by Gould and White [21], while the study of the perception of the world map using sketch methods was the subject of a global project supported by the National Geographic Society [13,22]. The image of the regions of the world among Czech students was studied by Polonsky et al. [23], the perception of Europe from a non-European perspective was investigated by researchers within the Eurobroadmap project [24], while the perception of Europe among its inhabitants, among others, by Padło [25]. The method of mental maps was applied not only in geography and urban planning. It was also used, among others, in research on communities of excluded people [26,27], perception of security in the cities [28], human life cycles [29], as well as in research on the quality of education [30] or, more broadly, in children’s studies, linguistics, psychology, sociology, etc. [31]. Besides of the way in which we construct the imagination of the world, the research of mental maps has focused, among others, on cartometric differences between the image and the real world [32,33]. The multitude and variety of fields in which this research tool is used proves its universality.

Although the term "mental map" is understood broadly [34,35], the meaning used in this study corresponds to the subjective representation of the real map. Various methods are used to obtain a feedback image: mental maps are often created with the use of verbal methods, such as interviews, focus studies [20,36,37] or building blocks, intended to reduce the differences in the respondents’ artistic abilities [38].

From the perspective of obtaining a spatial image representing an individual or being a cognitive map of a group, drawing a sketch map [39,40], understood as a freehand sketch (potentially computer-supported), or labeling an already existing map seem to be the most appropriate methods [20]. Although the quality of mapping is influenced by many factors, and the method itself is subject to criticism [41] and raises interpretation issues [42], the role of this type of research in social perception does not decrease [35], especially that, although cognitive maps have a unique, individual character, they also contain parts common to larger populations [43].

Rivers are an important element of research on landscape perception [44], as already noted by Lynch, who defined their importance as edges in the perception of urban space [19]. Treating rivers as an element of the landscape forces focusing on the local scale in the study of their perception. Research on the social perception of clarity and color of water on several sections of New Zealand rivers in the context of their recreational values [44] or the perception of the Chicago River greenway corridor by 3 categories of respondents [45] fit into this locality. The research methods used, based on the questionnaire [44], hand-drawn landscape drawings [45] or (The Photo-Projective Method) (PPM) [46], focus on the study of river sections that are known and experienced for the respondents. A wider geographical scale is characteristic for e.g. anthropological research among the peoples of Siberia, in which rivers represent the axes along which structured spatial information is organized in the minds of the local populations [47,48], while there is basically no research on the perception of the course of rivers using the sketch map method. One of the few works in this area are the research on children’s understanding of the river system [49] and the works of Angiel [50] on the perception of the course of the Vistula. The author notices large discrepancies in the drawing of the river course and the lack of its knowledge even among students living directly on its banks. The respondents treat it more as a symbol than a geographical object. In other studies, the author draws attention to the broad spectrum of symbolical values that the respondents bestow on the Vistula: national, urban, natural, cultural, recreational and sentimental ones [51].

## Materials and methods

**Fig 1:**
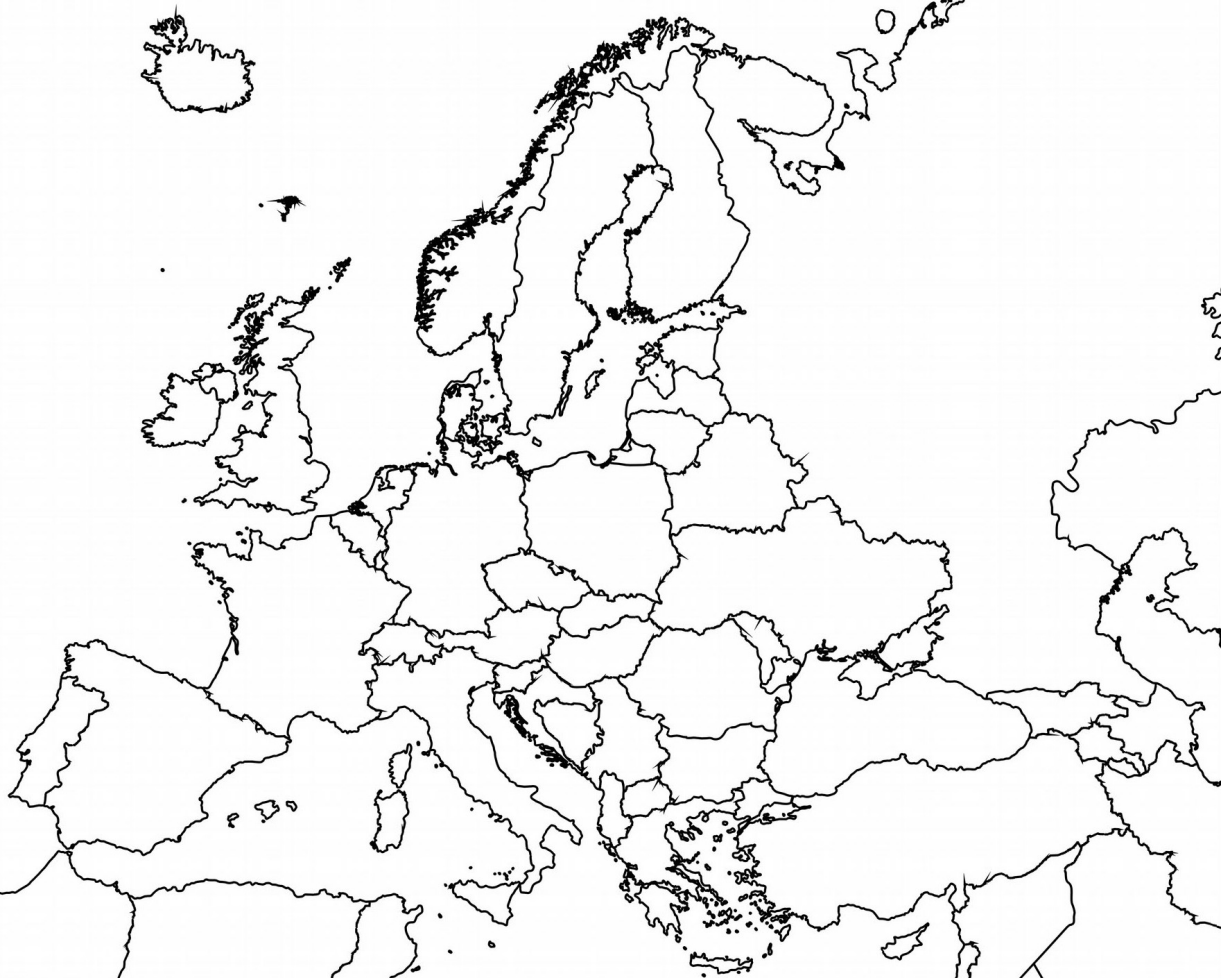
Map of Europe included in the questionnaire. The map was prepared using political boundaries from the dataset of thematicmapping.org.

The research was conducted in seven European cities: Bălţi (Moldova), Bern (Switzerland), Cork (Ireland), Krakow (Poland), Niš (Serbia), Porto (Portugal) and Uppsala (Sweden). The selection of the cities was based on their multifunctional character and high rank in the settlement hierarchy of particular countries. In order to avoid the potential impact of metropolitan character of the city on perception of the space by its inhabitants, the capital cities were excluded from the study (except of Bern, assuming that in case of Switzerland–Zurich is the city performing metropolitan functions). The research covered students from 38 classes in 19 selected schools in 2005–2007 and again students from 34 classes in 17 schools in the same cities in 2015–2018. Two or three schools were selected in each city (except of Bern, where the research was conducted in one school only). The respondents were the students of penultimate classes of general secondary schools which completion enabled continuation of studies in high schools. The study covered mostly respondents in the age between 16 and 18 years (94% of respondents were of such age). The age differences between the respondents derived mostly from different education systems and the related differences in the age of commencing education. Such selection of respondents enabled comparing social groups of similar educational competences, thus mitigating the differentiating impact of knowledge and age on perception of the space. The respondents were asked to perform the following exercise: „Below is a map of Europe. Put down in the right place Danube River”. The contour map was of 15x12 cm dimensions and the state borders were presented on it (Fig 1).

The exercise was a part of a broader questionnaire; the respondents had 25 minutes to fill it. The outcome of the research comprised of 806 surveys in the first research period, and 357 of them (44%) included the Danube river drawn in the maps. In the second period, 771 surveys were obtained, with Danube present in 349 (45%) of them. The distribution of frequency of drawing the Danube in particular cities has been shown in Table 1. The research was conducted at the presence of one of the authors, what enabled con-trolling the separate character of performing the task by the respondents. The contour of the political map enabled the respondents to use the distribution of the countries for more precise positioning of the river and placing it in the political and cultural context. It was assumed that respondents on this level of education possessed enough geographical competences to distinguish the contours of the states, what they proved in other parts of the questionnaires [52]. In order to present the group imagination of the course of the Danube river, the method of non-parametric kernel density estimators was applied, giving the synthetic picture of imagination maps of various groups of respondents. The paper questionnaires were transformed to digital form through scanning, using standard office equipment. The content of the maps was redrawn in Corel Draw X5 program: lines representing the course of the Danube river were transformed to cdr vector layer. Later on, the raw scans were provided with georeferences. ESRI ArcGIS software was used to change the background projection to Lambert azimuthal equal-area projection (LAEA, code EPSG 3035) used in Europe. Vector files from Corel Draw were imported, using *.dxf format (CAD). Later on, 50 control points identical for both maps were identified and found using the Spatial Adjustment tool. The maps were matched by applying projective transformation. The average RMSE square error at the surface of 16 kilometers was obtained, being a satisfying value for a map covering the whole Europe. At the end of this stage of the works, the contours of political borders were removed from the questionnaire maps and only the linear course of the Danube was left. This resulted in obtaining consistent picture of the outcome of questionnaires in the form of shapefile vector files, organized according to the cities which the respondents came from.

**Table 1:** Share of questionnaires including drawn course of the Danube in particular cities (%).

	Bălţi	Bern	Cork	Krakow	Niš	Porto	Uppsala
**2005–2007**	33,9	60,0	27,9	65,9	56,3	22,0	59,6
**2016–2018**	31,5	72,3	58,1	54,5	63,5	31,1	28,1

**Fig 2:**
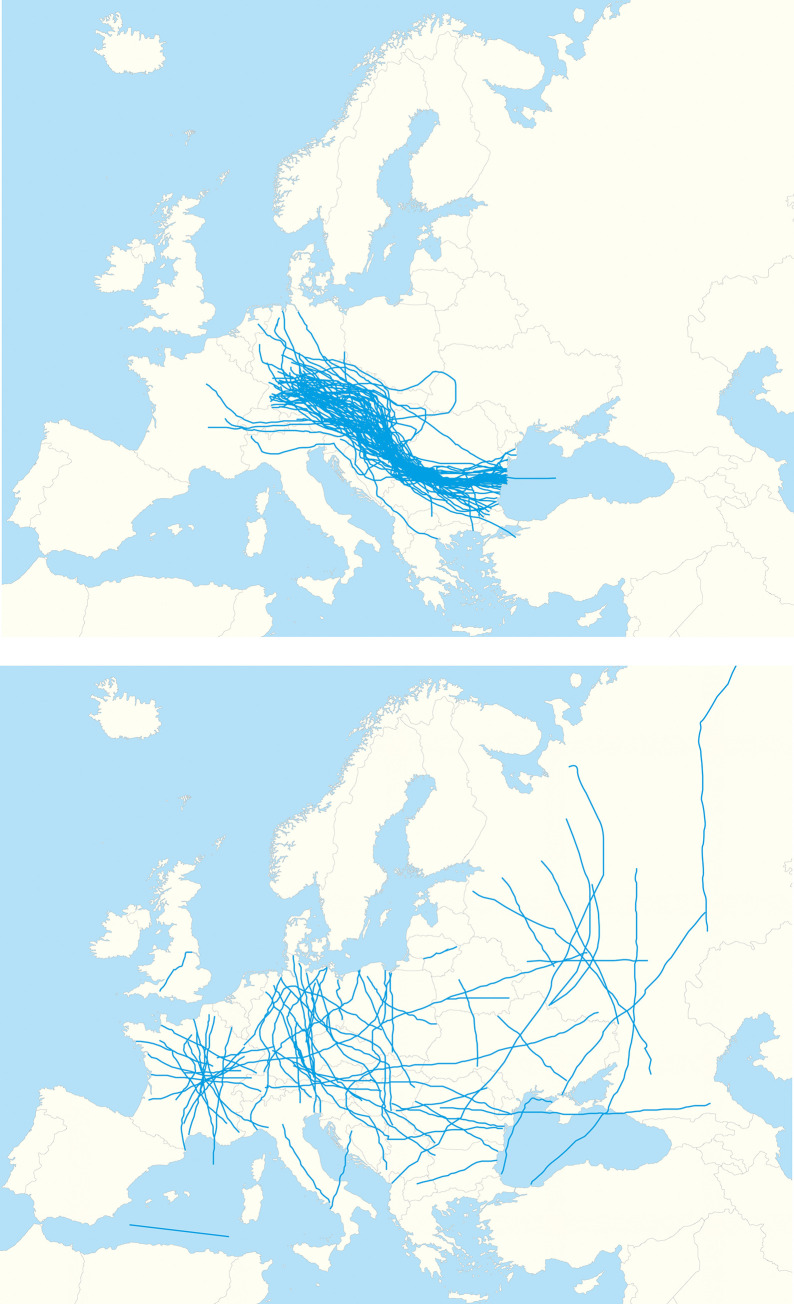
Cases of aggregated sketch maps for Niš in 2006 (left) and Uppsala in 2007 (right). The map was prepared using political boundaries from the dataset of thematicmapping.org.

Already the comparison of the drawn lines with the course of the Danube river would enable in-depth analysis of feedback of respondents from various cities (Fig 2). It was however decided to quantify the outcome, using the method of non-parametric density estimators (kernel analysis). In this method, a continuous surface is generated along every line, while the intensity of the phenomena decreases along with the distance, reaching zero at the distance determined by the value of the radius fixed by the ’kernel’ tool. In this study, the default radius value was kept. The kernel density function matches the radius value to the analyzed set of points or lines [53]. Thus, the same set of values was not maintained for each examined case, due to untypical concentration of lines in cases of Krakow and Niš. The intentional visual effect showing the spatial distribution of the course of the Danube, drawn by students in particular cities, was obtained through presentation of the density of the lines in the form of isoline map. The values were grouped into 7 value classes using the equal intervals method, keeping the class signatures common for all cities. The maps show the responses of respondents from 2005–2007 and 2016–2018. A mean centre was also generated for the responses from the respective periods. The placement of maps made with exactly the same cartographic method al-lowed for noticing changes in the perception of the Danube among students. This made it easier to draw substantive conclusions from the obtained research material.

## Results

**Fig 3:**
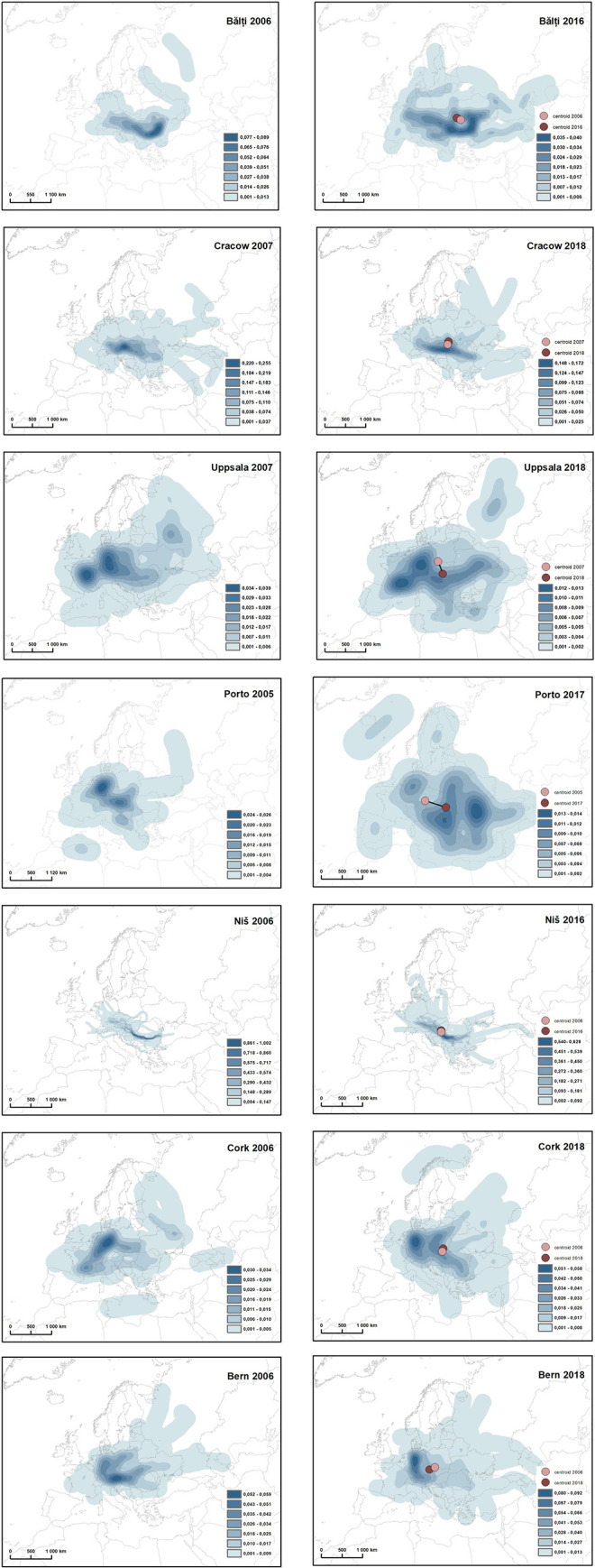
Perception of Danube by respondents from selected cities. The map was prepared using political boundaries from the dataset of thematicmapping.org.

Perception of the course of the Danube is very much differentiated, depending on respondents’ places of living. Already the share of respondents who took up the task (Table 1) shows that it brought much difficulty to students from Cork, Porto and Uppsala–cities located most remotely from the Danube and from Bălţi, (which is surprising in the context of Moldova’s access to the Danube). It should be noted, however, that students from Moldova, compared to others, relatively often did not provide answers. The analysis of generated maps indicated that in both cases the imagination of the course of the Danube is much different from the actual one, especially in Western European countries (Fig 3). On the other hands, in the Danube countries (Serbia and Moldova; the last one having only ca. 2 km access to one of the arms of the Danube delta) it is much closer to the actual course, although the cities of respondents’ residence are located in significant distance from the river. The particularly high awareness of the river course is presented by students from Niš, who draw it almost perfectly along its whole course what is clearly visible in Fig 3. The Danube is treated in Serbia as the symbol of the country [54] and a kind of a ’window to the world’. Over ages it marked in nowadays Serbia the limits of Roman Empire, Ottoman Empire or the border between the Austro-Hungarian Empire and the Kingdom of Serbia. The importance of the Danube translates to its presence in formal education and, hence, good recognition among the respondents. In case of students from Bălţi, the mouth of the river and its border section between Romania and Bulgaria are particularly well recognized. This is most probably related to the huge economical and geopolitical importance of the mouth of the river to Moldova. Also the students from Krakow recognize well the Danube basin area, yet they indicate the river course in a much less correct way.

In case of respondents from Western European cities, the recognition of the Danube course is very poor, except of Bern. The wrong answers show significant Westcentrism, visible in marking the Danube course in Germany and, to less extent, France, what can be particularly well noticed in the drawings of respondents from Uppsala (Fig 3). This is i.a. related to poor knowledge of Eastern Europe [25], limited experience of place, shared by inhabitants of this part of the continent in comparison to the Western Europe, or, eventually, the stronger enrooting of the Danube in the culture of the West, in particular in its German part. Moreover, many respondents searched for the mouth of the Danube in the North Sea, what may suggest that its course is mistaken for the course of the Rhine, another big European river of much higher economic importance. The tendency to “drive” the Danube towards the place of residence is also visible–it is a common phenomenon occurring when assessing the location of objects in the space [52].

Cases of maps generated by respondents from Bern and Krakow are also interesting: there, besides of concentration of lines determining the imagined course of the Danube (superficial–wrong and linear–accordant to the reality), more intensive concentration of indications in the North-East of Austria, near Vienna, is visible. This almost punctual accordance between the course of the river and its placing results from its strong symbolical association with the Austrian capital city. Much less concentration can be found nearby Budapest, thus corroborating the hypothesis of Westcentrism in space perception. In spite of the visible Westcentrism, the map presenting the generalized results of perception of the Danube by the respondents (Fig 3) matches its actual course quite well. This can be particularly well noticed on the Austrian–Hungarian stretch and along the Romanian–Bulgarian border, what to some extent supports the previous re-marks.

Thus, the nature of the river course drawn by the respondents assumes three types of compliance:

1. linear: the course corresponds to the actual course of the river. Characteristic for respondents from Niš (Serbia) and Bălţi (Moldova) for whom the Danube is a cultural value or a window to the world;
2. punctual: with a clear concentration of the marked course in tourist cities associated with the presence of the river, mainly Vienna and Budapest. Characteristic for respondents from Krakow and Bern;
3. superficial: the superficial nature of the created image, resulting from the scattered, imprecise placement of the river, effecting from shortage of knowledge about its course. Characteristic for respondents from Western Europe.

The centroids marking the average point of the drawn lines of the Danube differ significantly between the compared years only in two cases. In the case of respondents from Uppsala and Porto, who marked the river’s course relatively less accurately, their center of gravity shifted to the east, which can probably be explained by the territorial expansion of the European Union in this direction. Another characteristic of the perception of the space is also visible: ethnocentrism, visible in the placement of centroids closer to the respondents’ place of residence. For Niš it is southern Hungary, for Bern the Czech Republic, for Krakow—Slovakia, and for Bălţi–Romania.

## Conclusions and discussion

Sketch maps allow for a more complete understanding of the level of geographical knowledge and the ways of its transformation and use [39], therefore their use in geographical research of symbolic objects, like the Danube, is justified. The article describes the differentiation of perception of the Danube from various cognitive perspectives among respondents representing the same age group and a comparable level of education, while different in terms of place of residence in the European space, especially in relation to the Danube.

The applied sketch map method clearly shows the differences in the location of the Danube, drawn on the map of Europe. The simplicity of the data collection method allowed to eliminate the limitations related to different artistic abilities of the respondents [55], and the use of the kernel analysis method made it possible to obtain a social image of the perception of the river course, similarly to the study of world map images, in which the fuzzy logic approach was applied in a similar way [56]. So far, little research has been conducted to present a synthetic image of perception of geographical objects [56], what is an additional value of the work.

Three types of concentration (superficial, punctual and linear) have been identified in the social perception of the river course. The course of the Danube, a river that is symbolic for Europe, generally remains a mystery to its inhabitants, what mainly concerns Western European respondents from cities located far away from the Danube. This may suggest that it might not be an object full of meaning and value for them, as the apologists of the Danube significance would imagine. They represent the superficial nature of the social perception of the river, characterized by its incorrect location, resulting from the lack of knowledge about its course, as well as the lack of experience of the river itself, which in turn result from, inter alia, the distance function; this is confirmed by the results of research on the influence of distance on the perception of space [57]. The large discrepancy between the drawn course of the river and the actual one seems to be much greater than in the case of state borders or the coastline of European countries [13,25], what may result from the overrepresentation of political maps not inclusding the river network in the public space. The punctual nature of perception, based on knowledge about the river or experiencing it through the prism of the cities that lie over it, is characteristic for respondents from Central Europe. They identify the river with Vienna or Budapest, popular cities that are popular tourist destinations of the region’s inhabitants. The experience of the city, important for the perception of geographical objects, reduces the knowledge of the river course to its urban section. Cities are reference points for drawing the course of the river. The image most consistent with reality, characterized by a linear perception of the river, is typical for respondents from countries of Central and Eastern Europe located on the Danube, especially from Serbia, for whom it is the backbone of the country and a window to the world.

It is surprising that the perception of the Danube course has not changed over more than a dozen years after the European Union’s expansion to the East and the related better understanding of Central and Eastern Europe. The centroid has shifted slightly, indicating that the Danube in social perception has shifted more to the East, but the types of social perception of the river have not changed during this period. This calls into question the uncritical treatment of the Danube as a symbol of reconciliation between the East and the West, and, in a broader context, raises the question about the reasons for the low importance of the river in the perception of the West.

The pan-European spirit which the river is expected to personify seems to be present rather in Eastern than in Western Europe, and in particular in countries located on the Danube. The respondents from the western part of Europe often draw the Danube in areas recalling the course of the Rhine, suggesting that from their point of view, the Rhine is actually the most important river in Europe. In that context, the words of Magris from 30 years ago, when the iron curtain was still in place (’the sensation of travelling eastward across Europe, from the world of the Rhine to the world of the Danube, from Europe to non-Europe’ [8] seem still actual and even the integration processes in Europe have not changed this situation so far.

Despite the concerns of some researchers that the mental map metaphor is a misleading analogy to the representation of geographical knowledge [58], the results of the work lead to a fairly obvious conclusion that building the meaning of symbolic places should be based on education and experiencing these places.

At the same time, research on the perception of space in a dynamic approach seems to be a good direction in understanding the changing world, and the applied method of perception of linear objects looks promising for further research on the images of geographical space.
